# 

**Published:** 1916-03-11

**Authors:** 


### Annual Meetings.

### Of Special Interest.

SEAMEN'S HOSPITAL, GREENWICH.—Tuesday, March 14, 1916, at 3 P.M., at Prince's Restaurant, Piccadilly, Major-General F. L. Lessard, C.B., Canadian Expeditionary Force, in the chair. Tea will be served in the restaurant at 4.30 P.M.

**The speakers will be**:—The Right Hon. Lord Devonport (Chairman of the Port of London Authority) and the Right Hon. Andrew Fisher (High Commissioner for the Commonwealth of Australia).

LONDON TEMPERANCE HOSPITAL, HAMPSTEAD ROAD, N.W.—Wednesday, March 22, 1916, in the Out-Patient Hall of the hospital, at 5 P.M., the Marquis of Lincolnshire, K.G., in the chair.

**The speakers will be:**—The Right Hon. the Bishop of Willesden, D.D., the Hon. Lady Barlow, Admiral Sir G. King-Hall, K.C.B., J. Porter Parkinson, Esq., M.D., M.R.C.P., Arthur Evans, Esq., M.S., F.R.C.S., the Right Hon. Sir T. Vezey Strong, K.C.V.O. (Chairman of the Board of Management), Henry Holloway, Esq., J.P. (Treasurer), and others.

CANCER HOSPITAL (FREE), FULHAM ROAD, S.W.—Monday, March 13, 1916, at 4 P.M., the Right Hon. the Earl of Northbrook (President) in the chair.

GREAT NORTHERN CENTRAL HOSPITAL, HOLLOWAY ROAD, N.—Thursday, March 16, 1916, in the Board-room of the hospital, at 5 P.M.

### The Recruitment of Doctors in England and Wales.

As we go to press we have received the following notice from the Central Medical War Committee :—" We are officially informed by the War Office that, in accordance with their previous announcements, doctors who have undertaken to accept a commission in the Royal Army Medical Corps (if offered one) will not be taken for general service; and, therefore, that any doctor in England and Wales who (whether attested under the Derby scheme or not) has enrolled under the scheme of the Central Medical War Committee, or has offered his services in the Royal Army Medical Corps direct to the War Office, should, if he receives a notice paper from a recruiting officer calling him up (whether by reason of attestation or under the provisions of the Military Service Act), return it to the recruiting officer, together with his certificate of enrolment, or the War Office acknowledgment of provisional acceptance, as the case may be, and the notice will then be cancelled and the practitioner remain in the Reserve until selected for a commission in the Royal Army Medical Corps. Doctors will not be called up, whether by reason of attestation or under the Military Service Act, until after March 31, 1916. Doctors in England and Wales who have not undertaken to accept a commission (if offered one) in the Royal Army Medical Corps will, when called up (whether by reason of attestation or under the Military Service Act), have the same rights of appeal for exemption as men who are not doctors; but all cases coming before the Central Tribunal will be decided by that tribunal after receiving advice from the representative committee of the medical profession specially recognised for the purpose. Analogous arrangements will obtain in respect of doctors in Scotland."

### Passing Events and Topics.

### Treatment of Sick War-prisoners in Switzerland.

ACCORDING to Swiss journals, the negotiations for accommodating sick prisoners of war in hospitals in Switzerland are now complete. It is understood that 1,000 French prisoners will be treated at hospitals at Leysin, and the same number of German prisoners at Davos.

### The New Red Cross Stationery.

A RED CROSS envelope, the principal feature of which is a design by Mr. John S. Sargent, is being placed on sale for the benefit of the funds of the British Red Cross Society. The design displays two figures, a nurse assisting a wounded soldier from a stretcher, against a Red Cross. The idea of the envelope recalls the abortive attempts of past years to introduce the somewhat similar hospital stamps into this country, where, it is curious to recall, they have never enjoyed the popularity, amounting to an immense vogue, attained by similar designs abroad, especially in Germany. After all, there is not much difficulty in understanding why people refused to plaster their envelopes with symbolical designs which were not recognised by the Post Office, and while often ugly, were invariably irrelevant in themselves; but an envelope, something that can be used, should receive a warmer welcome; and the notion shows an advance in common sense that leads one to hope it may prove popular. At all events it provides the latest instance of ingenuity among the many which have been devised to raise money for charities directly dependent for their existence on the war. From Red Cross envelopes to Red Cross notepaper is but a step, and we understand that the step has already been taken. The designs for notepaper have been made by numerous artists, and the stationery can be obtained in penny packets. Messrs. Millington and Sons, of Budge Row, E.C., are the makers and publishers, from whom the envelopes and notepaper can be obtained.

Rates of Subscription: Twelve months, 6/6; Six months, 4/-; Three months, 2/-, post free to any address in the United Kingdom. Abroad, Twelve months, 8/8; Six months, 5/-; Three months, 2/6, post free.—Address The Subscription Dept., "The Hospital." The Hospital Building, 28/29 Southampton Street, Strand, London, W.C.

